# Association of Clinical Pathologists, Wessex Branch

**Published:** 1983-04

**Authors:** 


### Association of Clinical Pathologists, Wessex Branch

Abstracts of Papers given at the Autumn Meeting held at the Postgraduate Medical Centre, Bath, 23rd November 1982

### CONGO 'BLUE' STAINING: A HISTOCHEMICAL PROBLEM

**J. D. Davies and S. L. Mera, Department of Pathology, University of Bristol, Bristol**

Congo Red has long been used both *in vivo* and *in vitro* to stain human amyloid. It is also recognised histologically as an elastic fibre dye. Apart from staining cellulose, Congo Red is well known as an indicator, turning blue in increasingly acid aqueous solutions between pH 5 and 3. Conventional Congo Red staining of amyloid employs differentiation in alkaline alcoholic solutions. Differentiation of Congo Red-stained sections in 1% hydrochloric acid – ethanol leads to blue staining of elastic fibres, whereas amyloid remains red as in the conventional treatment.

Modifications of the dying procedure have given insight into the structure of elastic fibres and their interaction with Congo Red. Dye bath manipulations using strong salt solutions, 8M urea and Dimethylformamide indicate that van der Waals forces, hydrophobic and hydrogen bonding mechanisms are responsible for the dye-elastic and dye-amyloid binding.

The effects of basic and acidic amino acids upon Congo Red, the modulation of staining reactions by anionic and cationic detergents, and the effect of histochemical blocking of carboxy and amino tissue radicles, indicate that anionic groups are responsible for Congo 'blue' staining of elastic fibres. Such anions may be situated in background matrix, the elastic-associated microfibrillary glycoproteins, or in the elastin fibrils.

### RESISTANT ORAL STREPTOCOCCI AFTER 2-DOSE AMOXYCILLIN PROPHYLAXIS

**P. J. Southall, N. J. Mahy, R. M. Davies and D. C. E. Speller,**

The ability of short-course amoxycillin prophylaxis (suitable for dental operations in patients susceptible to infective endocarditis) to evoke resistant oral streptococci was tested by repeated administration at weekly intervals, on up to five occasions to 12 healthy dental students. None of the subjects showed resistant streptococci in plaque or saliva before amoxycillin administration. They were detected in one volunteer after one administration and in all after five administrations; the median number of administrations before resistance was observed was four. All the resistant isolates that were identified were *Streptococcus sanguis* (non-dextran-producing); amoxycillin minimum inhibitory concentrations ranged from 1.0–16.0 mg/l (median 8 mg/l). These findings must be applied with caution to dental practice in patients, but it is suggested that if all at-risk procedures cannot be accomplished in one treatment session an alternative regimen should cover further dental treatment within three months.

### LYMPH-NODAL HAEMANGIOMATOIDS

**M. F. Lott and J. D. Davies, Department of Pathology, University of Bristol, Bristol**

Lymph nodes were systematically examined by dissecting microscopy of 5 µm paraffin sections at a magnification of ×10. Lymph nodes (1073) from 250 patients were scrutinised. A search for vascular abnormalities yielded, after exclusions on the grounds of artefactual abnormalities, changes which fell into three groups. Four cases of miscellaneous vascular abnormality including hilar musculo-vascular change, post-inflammatory sclerosis and two other ischaemic lesions were identified. Pan-nodal Vascular Dilatation, defined as vasodilatation in all nodal zones and in the perinodal connective tissue, was found in 132 nodes from 55 of the 250 patients. Two cases of lymph nodal haemangiomatoids were examined, one only being discovered in the retrospective survey. In contrast to Pan-nodal Vascular Dilatation, nodal haemangiomatoids were uncommon lesions which were limited to a single lymph node in a sample of the regional nodes. Pan-nodal Vascular Dilatation was more common, affected more nodes in an affected chain than would be expected by chance, and particularly affected lymph nodes draining ulcerative colitis. Nodal haemangiomatoids were lesions localised in the lymph nodes, affected only a single node in the chain, and were associated with breast carcinoma in their vicinity. The observations suggest that Pan-nodal Vascular Dilatation is a reaction to a diffusable agent produced in inflammatory conditions, and that haemangiomatoids are a less common response to malignant disease.

---

### ASBESTOS RELATED DISEASES IN PLYMOUTH

**R. A. B. Drury, A. C. Hunt, W. L. H. Scarratt and Ruth M. Coles, Plymouth General Hospital and Medical Research Unit, H. M. Naval Base, Devonport, Plymouth**

---

The pathology of pleural fibrosis and malignant mesotheliomas in Plymouth has been investigated at autopsy. Pleural fibrosis was present in 22% of males exposed to asbestos and was localised as plaques, diffuse or mixed. Severe diffuse fibrosis may be disabling but plaques were often asymptomatic and radiologically negative. The duration and degree of asbestos exposure in dockyard workers was quantified and pleural fibrosis and mesothelioma both followed similar intermittent or moderate exposure.

Eighty-four men and one woman with malignant mesothelioma came to autopsy between 1972–82. Eighty-three were pleural, two peritoneal, almost all of diffuse type and associated with longstanding pleural fibrosis. Interstitial pulmonary fibrosis (asbestosis) was absent in 66% of men with pleural mesothelioma and when present was usually slight, never severe. The histological cell type of malignant mesotheliomas was of pure epithelial (carcinomatous) or mesenchymal (sarcomatous) type in 59%, mixed in the rest. Metastases were found in 28% of cases, but were never sufficiently massive or numerous to cause death, which was due to the local direct spread of the tumour. Lymph node metastases were from mesotheliomas with a carcinomatous cell type but visceral metastases were usually from sarcomatous or mixed tumours. Comparisons of cell type with age and the severity of asbestos exposure suggested that carcinomatous mesotheliomas developed at an earlier age and were associated with heavier exposure than sarcomatous or mixed tumours. Safety measures can be expected to diminish asbestos-related pathology, but there was a mean interval of 41 years between first asbestos exposure and death which suggests that malignant mesotheliomas will be prevalent into the next century.

---

### A GOOD PROGNOSIS GROUP IN CHILDHOOD ACUTE LYMPHOBLASTIC LEUKAEMIA

**Robert J. Stockley, Patricia Y. Ahlquist, and Martin G. Mott, Department of Child Health, Royal Hospital for Sick Children, Bristol**

---

The records of 121 children presenting with acute lymphoblastic leukaemia between 1969 and 1982 were reviewed. The overall relapse free survival rape was 50%. However girls presenting with a total white blood cell count of $20 \times 10^9$/l or less (35% of all patients) had a particularly favourable prognosis with an 80% relapse free survival rate at 12 years. The difference in prognosis between the sexes is confirmed but this difference is confined to the low initial white cell count group. We suggest that girls in this group should be excluded from the more intensive arms of new treatment protocols.

---

### THE VALUE OF CATHETER URINE SPECIMENS

**Dr. A. J. Davies and Dr. K. J. Shroff, Microbiology Department, Southmead Hospital, Bristol**

---

Catheter urine specimens (C.S.U.'s) are commonly sent from patients just before removal of their urinary catheter. We felt it would be more logical to examine midstream urine specimens (M.S.U.'s) taken after the catheter was removed, when removal of the foreign body would remove the source of infection.

C.S.U.'s were taken from 72 patients with short term urinary catheter (average length of catheterisation 4.1 days) just before the catheter was removed. The patients were assessed to see whether this specimen influenced their management. Their subsequent urinary symptoms were followed, and daily M.S.U.'s compared with the initial C.S.U. to see if the initial C.S.U. gave an accurate indicatinn of the likelihood of infection when the catheter was removed.

No growth or nonsignificant growth showed in 38 patients (53%) on both the initial C.S.U. and later M.S.U., but 18 patients (25%) showed significant bacteruria on the C.S.U. but no growth of a nonsignificant growth on subsequent M.S.U.'s, Only one patient was given antimicrobial therapy as a consequence of the initial C.S.U.

It would appear that C.S.U.'s taken prior to the removal of urinary catheters are of limited value, and may give potentially misleading results.

